# Multi-Granularity Analysis of Brain Networks Assembled With Intra-Frequency and Cross-Frequency Phase Coupling for Human EEG After Stroke

**DOI:** 10.3389/fncom.2022.785397

**Published:** 2022-03-31

**Authors:** Bin Ren, Kun Yang, Li Zhu, Lang Hu, Tao Qiu, Wanzeng Kong, Jianhai Zhang

**Affiliations:** ^1^College of Computer Science, Hangzhou Dianzi University, Hangzhou, China; ^2^Key Laboratory of Brain Machine Collaborative Intelligence of Zhejiang Province, Hangzhou, China; ^3^Department of Neurology, Zhejiang Provincial Hospital of Chinese Medicine, Hangzhou, China

**Keywords:** stroke, cross-frequency coupling, functional connectivity, brain network, mental rotation

## Abstract

Evaluating the impact of stroke on the human brain based on electroencephalogram (EEG) remains a challenging problem. Previous studies are mainly analyzed within frequency bands. This article proposes a multi-granularity analysis framework, which uses multiple brain networks assembled with intra-frequency and cross-frequency phase-phase coupling to evaluate the stroke impact in temporal and spatial granularity. Through our experiments on the EEG data of 11 patients with left ischemic stroke and 11 healthy controls during the mental rotation task, we find that the brain information interaction is highly affected after stroke, especially in delta-related cross-frequency bands, such as delta-alpha, delta-low beta, and delta-high beta. Besides, the average phase synchronization index (PSI) of the right hemisphere between patients with stroke and controls has a significant difference, especially in delta-alpha (*p* = 0.0186 in the left-hand mental rotation task, *p* = 0.0166 in the right-hand mental rotation task), which shows that the non-lesion hemisphere of patients with stroke is also affected while it cannot be observed in intra-frequency bands. The graph theory analysis of the entire task stage reveals that the brain network of patients with stroke has a longer feature path length and smaller clustering coefficient. Besides, in the graph theory analysis of three sub-stags, the more stable significant difference between the two groups is emerging in the mental rotation sub-stage (500–800 ms). These findings demonstrate that the coupling between different frequency bands brings a new perspective to understanding the brain's cognitive process after stroke.

## 1. Introduction

Stroke is a kind of cerebrovascular disease affecting the whole world. In most countries, stroke is the leading cause of the disability of adults and hinders the daily routine of patients and their families (Donkor, 2018). In recent years, neuroimaging techniques, such as CT, positron emission computed tomography (PET), functional MRI (fMRI), are often used in clinical treatment and disease research to monitor the neurological function of patients with stroke and explore the plastic reorganization mechanism of the brain (Rossini et al., 2003). However, these techniques are usually not portable and very expensive. Electroencephalogram (EEG) is a convenient and non-invasive technology with a high temporal resolution, which is suitable for monitoring, prognosis, and evaluating stroke disease (Monge-Pereira et al., 2017).

Previous research has proposed several Quantitative EEG (QEEG) features to evaluate brain activity changes after stroke, such as delta/alpha power ratio (Schleiger et al., 2014; Finnigan et al., 2016), brain symmetry index (Sheorajpanday et al., 2009; Sebastian-Romagosa et al., 2020), and laterality coefficients (Park et al., 2016). Besides, nonlinear parameters are also used in stroke research, such as Lempel Ziv complexity, sample entropy (Liu et al., 2016), and nonlinear separate degree (Zeng et al., 2017). These features are mainly analyzed based on single channels and cannot reflect the functional interactions between different brain regions. Therefore, some researchers explored the characteristics of the brain network after stroke. For instance, Philips et al. (2017) constructed brain networks in the beta band based on EEG data of the intensive therapeutic intervention period and found graph theoretical metrics are significant biomarkers to evaluate stroke rehabilitation. Although current studies reveal that oscillations in intra-frequency bands are reliable tools for exploring brain abnormality after stroke, the oscillatory mechanisms between different frequency bands have not been clearly understood.

More and more studies have shown complex brain information interaction between different frequency bands, known as cross-frequency coupling (CFC). Several brain regions of human and non-human primates found CFC phenomena, such as the hippocampus, prefrontal cortex, and sensory cortex (Mormann et al., 2005; Canolty et al., 2006; Jensen and Colgin, 2007; Khamechian and Daliri, 2020). Besides, increasing researchers use CFC to analyze cognitive and perceptual processes. For example, Dimitriadis et al. (2015) explored the coupling between the theta band and alpha band in the frontal lobe, parietal lobe, and occipital lobe during mental arithmetic tasks. Davoudi et al. (2021b) found an important parieto-occipital alpha-gamma coupling mechanism to rapidly select features from visual working memory storage. In the meantime, CFC shows advances in understanding the impact of many neurological diseases, including Alzheimer's disease (Cai et al., 2018), epilepsy (Jacobs et al., 2018; Yu et al., 2020), social anxiety disease (Poppelaars et al., 2018), and multiple sclerosis (Ahmadi et al., 2019). For instance, Jacobs et al. (2018) extracted cross-frequency phase-amplitude coupling features to predict seizures, and Yu et al. (2020) constructed the cross-frequency phase-phase coupling from seizure interval to seizure period. In human stroke-related studies of EEG signals, some studies focused on the CFC between EEG and other physiological signals, such as EMG (Xie et al., 2021) and cerebral blood flow velocity (Liu et al., 2019). Other studies investigated the CFC of EEG signals. For instance, based on the EEG data of the upper limb movement experiment, the effective network of 5 predefined motor cortex areas was constructed by dynamic causal modeling (DCM) to identify the biomarkers for classifying the patients' recovery state (Larsen et al., 2018). In addition, the DCM was utilized to investigate intra-cortex and inter-cortex effective connectivity of the 3 motor cortex areas in the intra-frequency and cross-frequency bands during the precision grip task in the stroke acute and sub-acute phase (Chen et al., 2017). In summary, the existing related studies on the cross-frequency analysis of EEG signals in patients with stroke mainly focus on the motor cortex during motor executive tasks. However, it may result in the obtained information being limited since it may lose potential information interactions between different brain regions. Moreover, the network information interaction of patients with stroke during the motor imagery process, which reveals the motor perception function after stroke, needs to be explored.

Mental rotation, a kind of motor imagery task, is conducive to restoring specific limbs' motor ability. Yan et al. (2013) constructed brain networks in beta bands based on the EEG data of patients with stroke and healthy controls during the mental rotation task and found significant alterations of the stroke brain in several temporal and spatial granularity. But this study has not explored the information interactions in cross-frequency bands. Previous analysis of healthy subjects in the mental rotation task found cross-frequency coupling between posterior parietal and frontal regions (Bertrand and Jerbi, 2009), which reveals the importance of CFC analysis in understanding the brain's mental rotation cognitive process. However, extracting effective features from CFC is more difficult than traditional intra-frequency coupling since the corresponding data is complicated and contains more hidden information. In addition, the cross-frequency features revealing the physiological mechanism of the brain requires to be deeply analyzed.

In this article, EEG data of patients with stroke and healthy controls during the mental rotation task is analyzed. A multi-granularity analysis framework is proposed, which uses multiple brain networks assembled with intra-frequency and cross-frequency phase coupling to evaluate the stroke impact in temporal and spatial granularity. In detail, spatial granularity includes analyzing the average phase synchronization index (PSI) at the whole brain area scale, hemisphere scale, and single-channel pairs scale. Besides, we also explore the brain networks in the temporal granularity, including graph theory analysis in the entire task stage and three sub-stages. The multi-granularity analysis shows that the brain information interaction of patients with stroke is highly affected in cross-frequency bands which demonstrates that the coupling of different frequency bands is an effective tool for exploring the impact of stroke.

## 2. Materials and Methods

### 2.1. Subjects and Recording

The data of this experiment is collected by Shanghai Jiaotong University and the Department of Neurology in the Fifth People's Hospital of Shanghai. The patients with stroke group consists of 11 patients with stroke, and the ischemic stroke lesions are in the left hemisphere of the brain. The healthy controls group consists of 11 subjects of similar age and has no history of neurological diseases or psychiatric disorders. A mental rotation task is conducted, and the visual stimulus materials are the pictures with different rotation angles of the left and right hands, 0°, 60°, 120°, 180°, 240°, 300°, respectively, recorded as S1, S2,..., S12. Stimulus pictures are randomly presented, and each subject needs to decide whether the presented picture is a left hand or right hand and make a corresponding keyboard response. Data is recorded by Brain Vision Recorder (Brain Products GmbH, Munich, Germany) with 30 EEG channels (FP1, FP2, F3, F4, C3, C4, P3, P4, O1, O2, F7, F8, T7, T8, P7, P8, FZ, CZ, PZ, Oz, FC1, FC2, CP1, CP2, FC5, FC6, CP5, CP6, TP9, TP10) and 2 EOG channels (HEOG, VEOG). In the experiment, the impedance is below 5 *kΩ*, and the sampling rate is 1,000 Hz. For more detailed information on the data, please refer to the original article (Yan et al., 2013).

### 2.2. EEG Pre-processing

**Figure 1:**
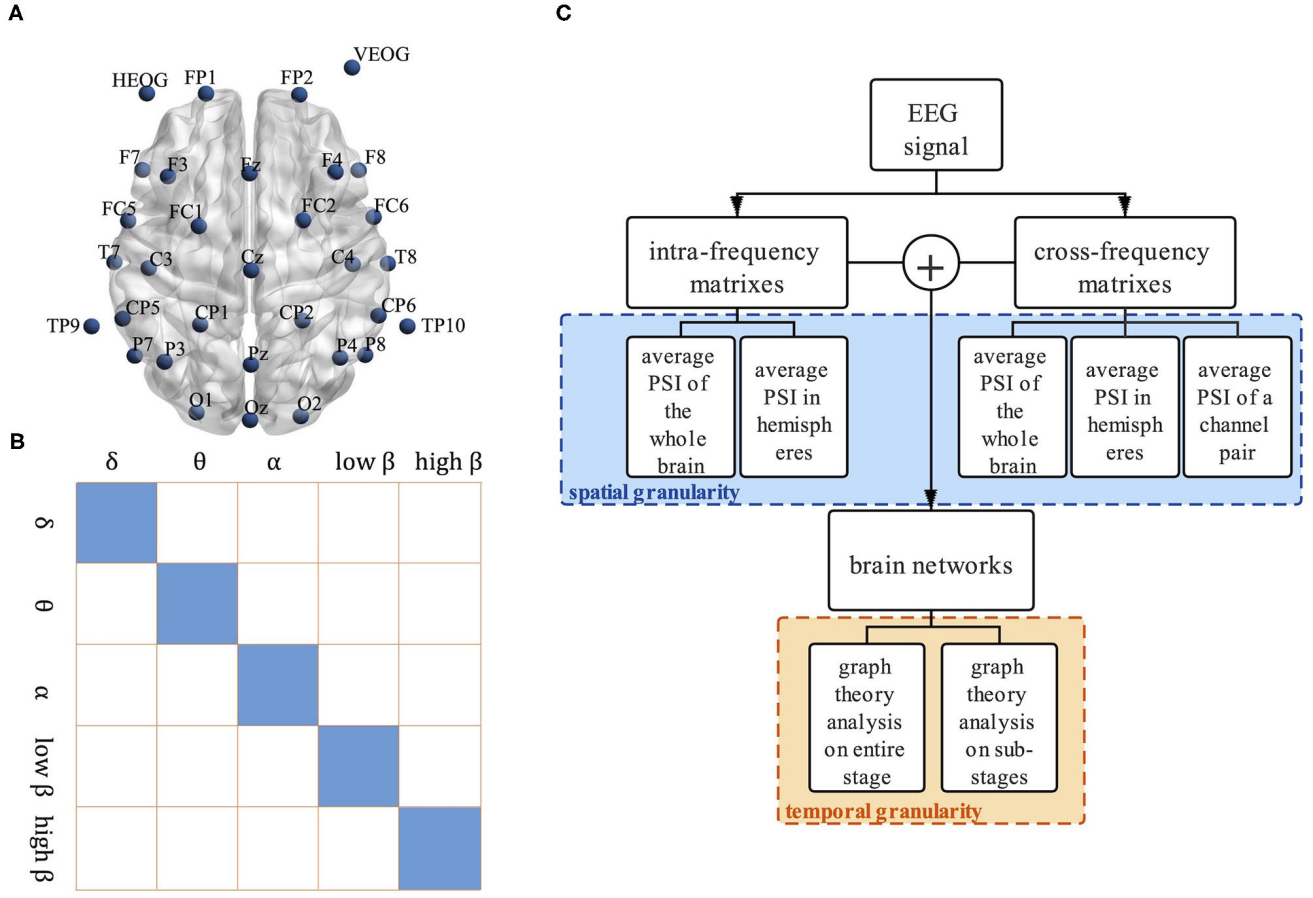
The framework of the proposed method. **(A)** The layout of channel location, **(B)** Visualization of structure between intra-frequency and cross-frequency connectivity matrices. Matrices show intra-frequency (diagonal tiles) and cross-frequency bands (off-diagonal tiles), and **(C)** Multi-granularity analysis framework.

First, the recorded EEG data is pre-processed. The original EEG data is filtered to 0.01–30 Hz by a band-pass filter, and the physiological artifacts such as Electrooculograms (EOGs) are removed by the Independent Component Analysis (ICA) method. After re-referenced to the channels TP9 and TP10, baseline correction is conducted. EEG data of 28 channels are segmented into the epochs of 1,000 ms starting from the onset of stimulation. The layout of the channel locations is shown in Figure 1A. Epochs with the correct keyboard responses are extracted and filtered to delta (δ, 0.1–4 hz), theta (θ, 4–8 hz), alpha (α, 8–12 hz), low beta (*low β*, 12–20 hz), and high beta (*high β*, 20 h–28 hz) bands. Previous studies showed that the cortical activation in each cognitive sub-stage of motor imagery altered after stroke (Yan et al., 2012). Therefore, EEG data of three sub-stages is extracted for the following analysis, including 0–300 ms visual stimulus perception sub-stage, 300–800 ms mental rotation sub-stage, and 800–1,000 ms response sub-stage.

### 2.3. Phase Synchronization Index

This study uses the n:m phase synchronization index to construct functional connectivity matrices of intra-frequency and cross-frequency bands (Cai et al., 2018). First, the original signal *x*(*t*) is calculated by Hilbert transformation, which is defined as


(1) $$\begin{array}{r} \tilde{x}\left(t\right)=\frac{1}{\pi }PV\int_{-\infty }^{\infty }\frac{x\left(\tau \right)}{t-\tau }d\tau \end{array}$$

where *PV* is the Cauchy principal value and $\tilde{x}\left(t\right)$ is the result of Hilbert transformation. The instantaneous phase ϕ(t) is calculated by


(2) $$\begin{array}{r} \phi \left(t\right)=\arctan\frac{\tilde{x}\left(t\right)}{x\left(t\right)} \end{array}$$

Then n:m PSI is calculated to measure the functional connectivity of two signals *x*_*f*_*m*__ and *x*_*f*_*n*__ with the mid-frequency *f*_*m*_ and *f*_*n*_ in time *T*,


(3) $$\begin{array}{r} PSI\left(x_{f_{m}},x_{f_{n}}\right)=\frac{1}{T}|\overset{T}{\underset{t=1}{\sum }}e^{j\left[\Delta \phi \left(x_{f_{m}},x_{f_{n}},t\right)\right]}| \end{array}$$

where the PSI is ranging in [0, 1] and Δϕ(*x*_*f*_*m*__, *x*_*f*_*n*__, *t*) is the instantaneous phase difference of the two signals,


(4) $$\begin{array}{r} \Delta \phi \left(x_{f_{m}},x_{f_{n}},t\right)=n\phi \left(x_{f_{m}},t\right)-m\phi \left(x_{f_{n}},t\right) \end{array}$$

When calculating the instantaneous phase difference of two signals in the same frequency band, *n* and *m* are set to 1. In the cross-frequency case, *n* and *m* are integers that need to meet *n* × *f*_*m*_ = *m* × *f*_*n*_. In our experiment, the ratio of the mid-frequency of the five bands is 1:3:5:8:12, so the *n* and *m* are set to minimum integers satisfying the requirement.

At last, we get 5 intra-frequency functional connectivity matrices and 10 cross-frequency functional connectivity matrices in each epoch. The size of each matrix is 28 × 28. A bigger symmetric matrix is shown in Figure 1B to clearly display the relationship between the intra-frequency and cross-frequency connectivity matrices, where the small tiles of the main diagonal represent the intra-frequency functional connectivity matrices while the off-diagonal tiles are the cross-frequency connectivity matrices.

### 2.4. Graph Theoretical Analysis

In this articles, two graph theoretical metrics are selected to describe the functional differentiation and integration ability of the brain. The characteristic path length is used to describe the global function integration and information interaction efficiency of the network. The characteristic path length of the whole network is the average value of the shortest path between any two nodes in the network, which is defined as


(5) $$\begin{array}{r} L=\frac{1}{N\left(N-1\right)}\underset{i\neq j}{\sum }l_{ij} \end{array}$$

where *N* is the number of nodes and *l*_*ij*_ is the shortest path length between node *i* and node *j*. The clustering coefficient is used to describe the functional differentiation ability of the network. The clustering coefficient of the whole network is the average value of the clustering coefficient of all nodes, which is defined as


(6) $$\begin{array}{r} C=\frac{1}{N}\overset{N}{\underset{n=1}{\sum }}C_{i} \end{array}$$

where *N* is the number of nodes and *C*_*i*_ is the clustering coefficient of each node. In detail, *C*_*i*_ is the average intensity of all triangles associated with each node, which is defined by the following formula,


(7) $$\begin{array}{r} C_{i}=\frac{2}{k_{i}\left(k_{i}-1\right)}\underset{j,k}{\sum }{\left({\tilde{w}}_{i,j}{\tilde{w}}_{j,k}{\tilde{w}}_{k,i}\right)}^{\frac{1}{3}} \end{array}$$

(8) $$\begin{array}{r} {\tilde{w}}_{i,j}=\frac{w_{i,j}}{\max\left(w\right)} \end{array}$$

where *w*_*i,j*_ is the weight scaled by the largest weight in the network and *k*_*i*_ is the degree of the node (Onnela et al., 2005).

### 2.5. Multi-Granularity Analysis

The multi-granularity analysis framework of the CFC is shown in Figure 1C. Analysis of Variance (ANOVA) is used to statistically analyze the PSI and graph theoretical metrics, and the significance level is set to 0.05.

After calculating the intra-frequency and cross-frequency functional connectivity matrices in every epoch, we get the mean functional connectivity matrices of every subject by averaging the mean functional connectivity matrices in 6 rotation angles in left-hand or right-hand motor imagery. Then we analyze the stroke patients' and controls' average PSI in spatial granularity, including the whole brain area scales, intra-left hemispheres scales, intra-right hemisphere scales, and inter-hemispheres scales. To explore the CFC in a further step, we also analyze the average PSI in every channel pair.

We analyze the brain networks in temporal granularity, including the entire task stage and three sub-stages. The brain networks are constructed by assembling intra-frequency and cross-frequency functional connectivity and then two graph theoretical metrics mentioned above are used for analysis.

## 3. Results

### 3.1. Analysis of Average PSI in Spatial Granularity

**Figure 2:**
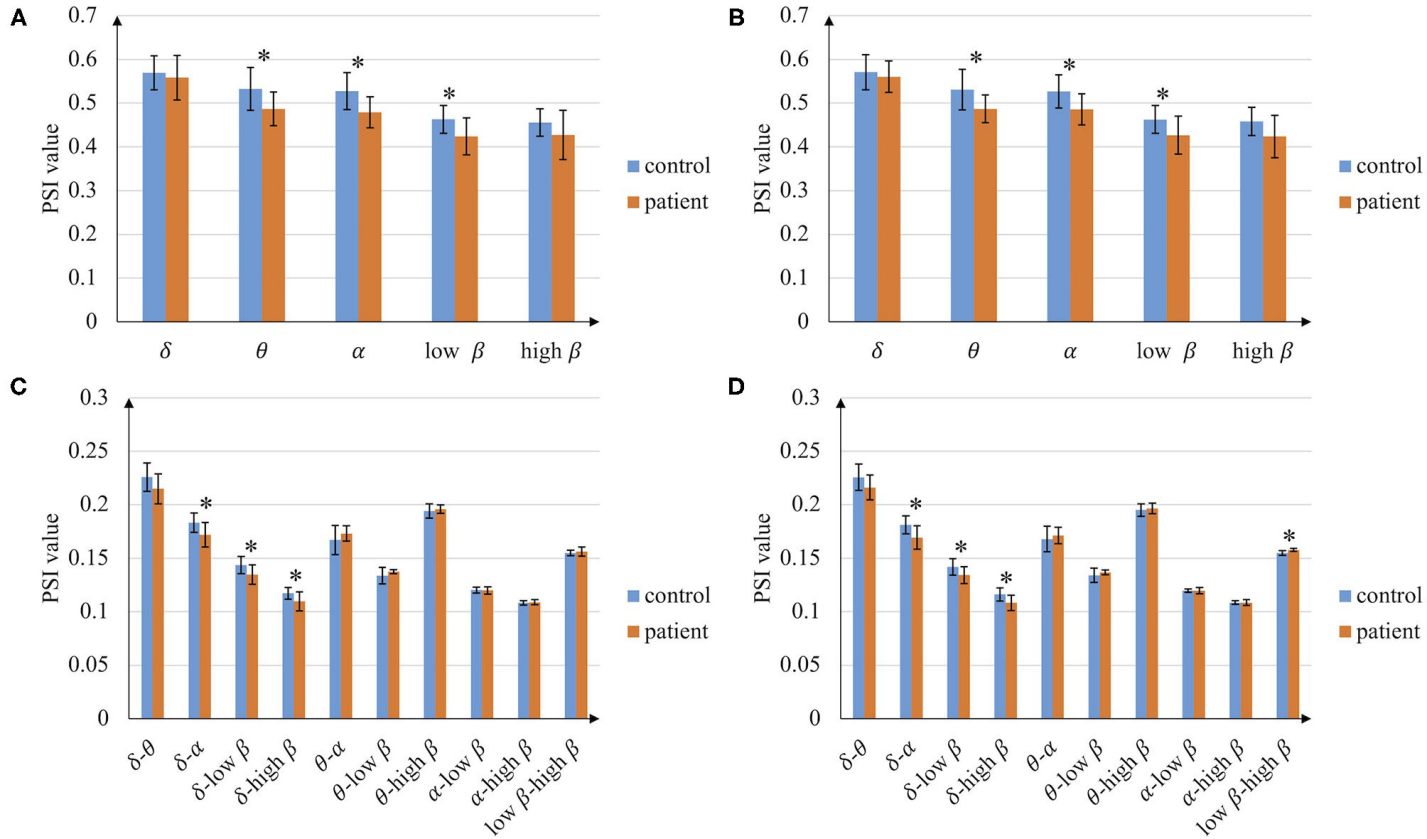
Average phase synchronization index (PSI) of the whole brain. **(A)** In intra-frequency bands during left-hand mental rotation task, **(B)** In intra-frequency bands during the right-hand mental task, **(C)** In cross-frequency bands during left-hand mental rotation task, and **(D)** In cross-frequency bands during right-hand mental rotation task (^*^p < 0.05).

The average PSI of patients with stroke and healthy controls are analyzed in the whole brain, hemisphere scale, and single channel pair. The average PSI of the whole brain is shown in Figure 2. In total, the average PSIs of the whole brain in several intra-frequency and cross-frequency bands show significant differences between patients with stroke and controls. PSI of patients with stroke is smaller than that of controls in intra-frequency bands, especially in theta, alpha, and low beta bands (*p* < 0.05). As for the cross-frequency bands, the average PSI of patients with stroke in delta-alpha, delta-low beta, and delta-high beta is smaller than that of controls (*p* < 0.05). The results between the left-hand and right-hand mental rotation tasks are similar in most frequency bands, while in the low beta-high beta cross-frequency band, the average PSI of patients with stroke is higher than that of controls in the right-hand mental rotation task (*p* < 0.05). According to the above result, the following analysis of PSI focuses on 3 intra-frequency bands (theta, alpha, and low beta) and 3 cross-frequency bands (delta-alpha, delta-low beta, and delta-high beta).

The average PSI is analyzed in the hemisphere scale, and the results are shown in Tables 1, 2. The left hemisphere of the brain is the lesioned hemisphere. It demonstrates that the average PSIs of lesion hemisphere and inter-hemisphere between patients with stroke and healthy controls are highly different in intra-frequency and cross-frequency bands (*p* < 0.05). More strikingly, we find that the cross-frequency PSI reveals richer information. The average PSI between patients with stroke and healthy controls in the non-lesion hemisphere also shows a significant difference(*p* < 0.05, Table 2) that can not be observed in intra-frequency bands.

**Table 1:** The *p*-values of average phase synchronization index (PSI) in hemispheres in intra-frequency bands.

**Brain regions**	**Theta**		**Alpha**		**Low beta**	
	**Left hand**	**Right band**	**Left hand**	**Right band**	**Left hand**	**Right band**
Intra-left hemisphere (lesion)	0.0049^**^	0.0015^**^	0.0048^**^	0.0129^*^	0.0253^*^	0.0143^*^
Intra-right hemisphere	0.8066	0.9254	0.7999	0.9103	0.5420	0.7746
Inter hemispheres	0.0163^*^	0.0152^*^	0.0026^**^	0.0095^**^	0.0261^*^	0.0545

* *p < 0.05*;

** *p < 0.01*.

**Table 2:** The *p*-values of average PSI in hemispheres in cross-frequency bands (especially the PSI of intra-right hemisphere between patients with stroke and healthy controls shows a significant difference).

**Brain regions**	**Delta-Alpha**		**Delta-Low beta**		**Delta-High beta**	
	**Left hand**	**Right band**	**Left hand**	**Right band**	**Left hand**	**Right band**
Intra-left hemisphere (lesion)	0.0642	0.0403^*^	0.0296^*^	0.0734	0.0520	0.0192^*^
Intra-right hemisphere	0.0186^*^	0.0166^*^	0.0310^*^	0.0440^*^	0.0657	0.0324^*^
Inter hemispheres	0.0219^*^	0.0082^**^	0.0520	0.0677	0.0320^*^	0.0205^*^

* *p < 0.05*;

** *p < 0.01*.

**Figure 3:**
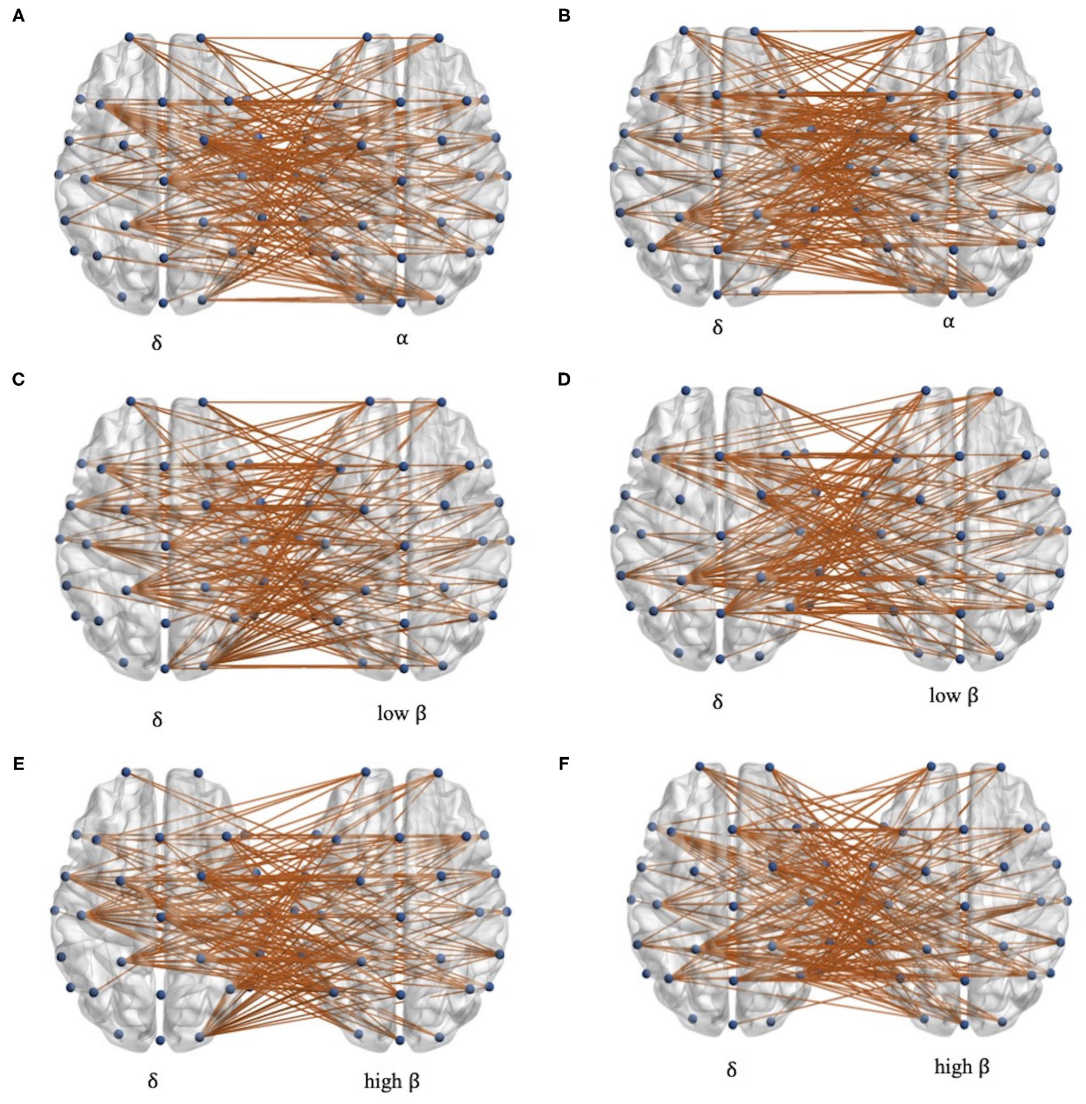
The distribution of channel pairs with the significant differences in each delta-alpha, delta-low beta, and delta-high beta band. **(A,C,E)** during left-hand mental rotation task and **(B,D,F)** during the right-hand mental rotation task.

To further explore the characteristics of cross-frequency functional connectivity, the PSI analysis of each channel pair in delta-alpha, delta-low beta, and delta-high beta cross-frequency bands is conducted. The channel pairs' average PSI with the significant difference (*p* < 0.05) between patients with stroke and controls are reserved, shown in Figure 3. In total, the channels with the significant difference in alpha, low beta, and high beta bands are located separately in the whole brain, while in the delta band, they are mainly located in the central area, frontal area, and parietal area. Besides, there are differences in the left-hand and right-hand mental rotation tasks in delta bands. For instance, the average PSI in the right occipital area between patients with stroke and healthy controls shows a significant difference during the left-hand mental rotation task, while the average PSI in the left parietal region between patients with stroke and healthy controls shows significant differences during right-hand mental rotation task.

### 3.2. Graph Theory Analysis in Temporal Granularity

We combine intra-frequency and cross-frequency functional connectivity to form a 56 × 56 functional connectivity matrix for the paired delta-alpha, delta-low beta, and delta-high beta, respectively. For instance, the 56 × 56 functional connectivity matrix for the paired delta and alpha bands are constructed from a 28 × 28 intra-frequency connectivity matrix within the delta band, a 28 × 28 intra-frequency connectivity matrix within the alpha band, a 28 × 28 cross-frequency connectivity matrix of delta-alpha, and a 28 × 28 cross-frequency connectivity transpose matrix of delta-alpha. Since the cross-frequency functional connectivity is smaller than intra-frequency functional connectivity, the same percentages of stronger functional connectivity are reserved (percentages between 20 and 80%, at 10% intervals) in each 28 × 28 matrix to obtain the critical functional connectivity of intra-frequency and cross-frequency simultaneously. Except for this manual threshold method, we also perform the automated threshold method, the orthogonal minimal spanning trees (OMST) topological filtering (Dimitriadis et al., 2017), to construct the brain networks for both intra-frequency and cross-frequency. After that, the brain networks assembled with two frequency bands with 56 nodes are obtained in each epoch, and then characteristic path length and clustering coefficient metrics are calculated. The average characteristic path length and clustering coefficient metrics of each subject in left-hand and right-hand mental rotation tasks are analyzed, and the results are as follows.

**Figure 4:**
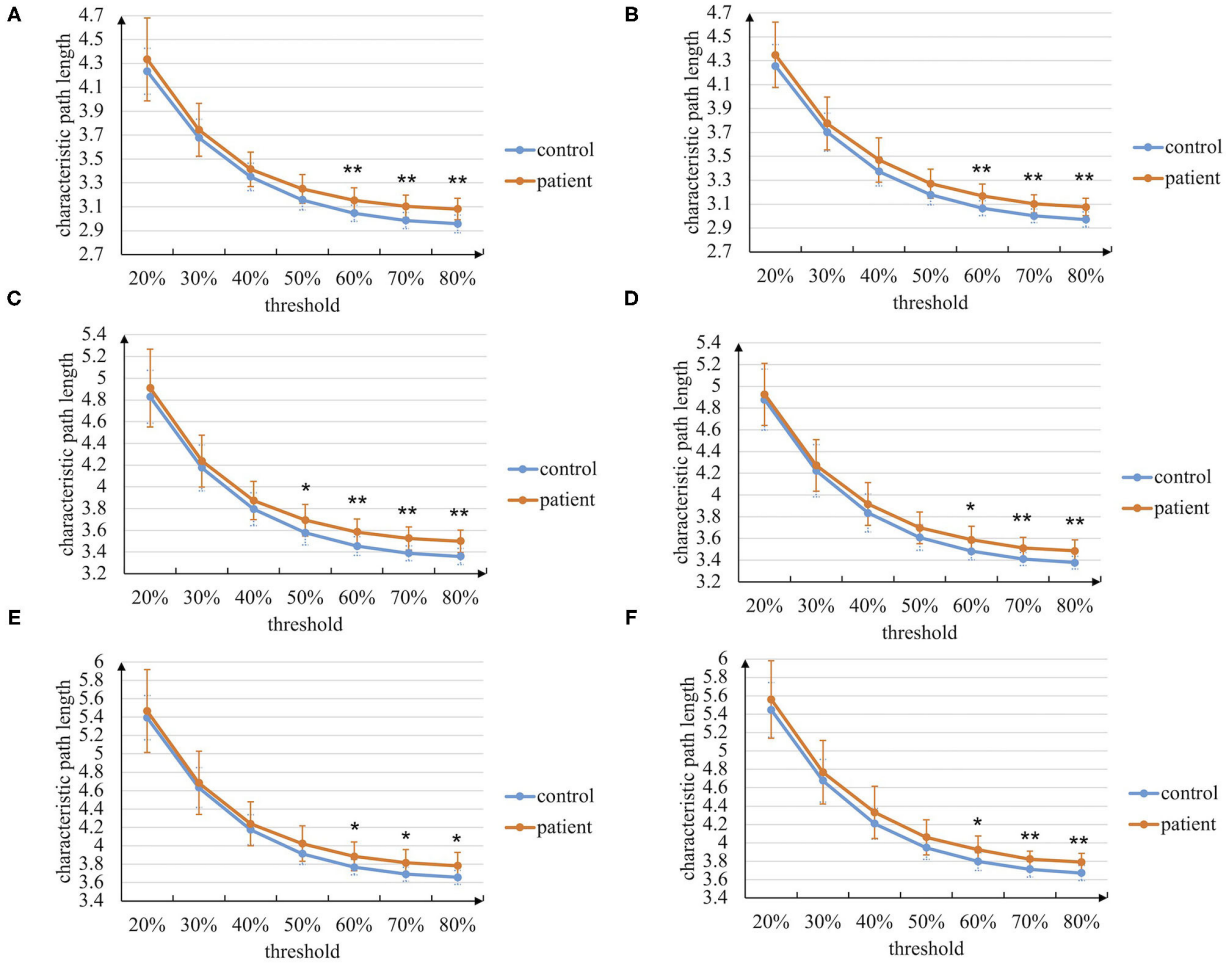
The characteristic path length of the brain networks based on the manual threshold method. **(A)** In paired delta-alpha band during left-hand mental rotation task. **(B)** In paired delta-alpha band during right-hand mental rotation task. **(C)** In paired delta-low beta band during left-hand mental rotation task. **(D)** In paired delta-low beta band during right-hand mental rotation task. **(E)** In paired delta-high beta band during left-hand mental rotation task. **(F)** In paired delta-high beta band during right-hand mental rotation task (* *p* < 0.05, ** *p* < 0.01).

**Figure 5:**
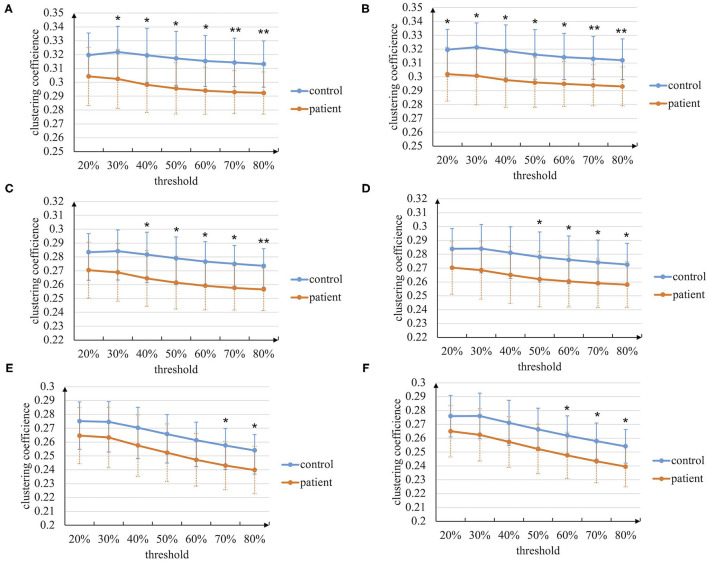
The clustering coefficient metric of the brain networks based on the manual threshold method. **(A)** In paired delta-alpha band during left-hand mental rotation task. **(B)** In paired delta-alpha band during right-hand mental rotation task. **(C)** In paired delta-low beta band during left-hand mental rotation task. **(D)** In paired delta-low beta band during right-hand mental rotation task. **(E)** In paired delta-high beta band during left-hand mental rotation task. **(F)** In paired delta-high beta band during right-hand mental rotation task (* *p* < 0.05, ** *p* < 0.01).

At first, the graph theory analysis of the brain networks during the entire task stage is conducted. The characteristic path length and clustering coefficient metrics based on the manual threshold selection method are shown in Figures 4, 5. In general, the characteristic path length of the brain networks of patients with stroke is greater than that of healthy controls, while the clustering coefficient is smaller than that of controls. This result indicates that the brain functional differentiation and integration ability of patients with stroke is significantly weaker. In more detail, when thresholds are set bigger than 60%, two graph theoretical metrics show significant differences (*p* < 0.05), and the characteristic path length is more sensitive to the threshold selection. Besides, the results in left-hand and right-hand mental rotation tasks are similar. These results reveal that brain networks constructed by intra-frequency and cross-frequency functional connectivity are effective tools to distinguish patients with stroke from healthy controls.

We also execute graph theory analysis of brain networks in three sub-stages by setting 70% as the threshold based on the above results. The ANOVA results of the two graph theoretical metrics are shown in Tables 3, 4. Regarding two graph theoretical metrics, the mental rotation sub-stage shows significant differences between patients with stroke and healthy controls in both metrics. In contrast, the other two sub-stage shows significant differences in only the clustering coefficient metric. Besides, the results under the mental rotation tasks of the left and right hands are similar.

**Table 3:** The *p*-values of characteristic path length metric of the brain networks based on the manual threshold method in three sub-stages (setting threshold as 70%).

**Stages**	**Delta-Alpha**		**Delta-Low beta**		**Delta-High beta**	
	**Left hand**	**Right band**	**Left hand**	**Right band**	**Left hand**	**Right band**
Visual stimulus perception	0.6120	0.0802	0.2091	0.0732	0.2728	0.0701
Mental rotation	0.0069^**^	0.0202^*^	0.0139^*^	0.0175^*^	0.0245^*^	0.0832
Response	0.1909	0.0928	0.2368	0.0692	0.3221	0.0630

* *p < 0.05*;

** *p < 0.01*.

**Table 4:** The *p*-values of clustering coefficient metric of the brain networks based on the manual threshold method in three sub-stages (setting threshold as 70%).

**Stages**	**Delta-Alpha**		**Delta-Low beta**		**Delta-High beta**	
	**Left hand**	**Right band**	**Left hand**	**Right band**	**Left hand**	**Right band**
Visual stimulus perception	0.0789	0.0121^*^	0.0012^**^	0.0058^**^	0.0455^*^	0.0020^**^
Mental rotation	0.0087^**^	0.0069^**^	0.0238^*^	0.0184^*^	0.0241^*^	0.0259^*^
Response	0.0074^**^	0.0045^**^	0.0007^**^	0.0032^**^	0.0182^*^	0.0058^**^

* *p < 0.05*;

** *p < 0.01*.

As for the brain networks topologically filtered based on OMST, the *p*-values of ANOVA of two metrics during the entire task stage and three sub-stages between patients with stroke and healthy controls are shown in Tables 5, 6. In general, the results are consistent with those using the manual threshold method. The characteristic path length and clustering coefficient metrics show significant differences between patients with stroke and healthy controls during the entire task stage (*p* < 0.05). Besides, the two metrics demonstrate more stable significant differences between the patients with stroke and healthy controls during the mental rotation sub-stage, while in the other two sub-stages, only the clustering coefficient metric shows a significant difference. Based on two different topological filtering methods, we analyze the characteristic path length and clustering coefficient metrics of the brain networks. The results are shown in Tables 7, 8, and the threshold of the manual threshold method is set as 70%. The characteristic path length metric of the OMST-based brain networks is larger than that of the brain networks filtered by the manual threshold method. In addition, the clustering coefficient metric of the OMST-based brain networks is also slightly larger.

**Table 5:** The *p*-values of the characteristic path length metric of the brain networks based on the orthogonal minimal spanning trees (OMST) topological filtering method in different stages.

**Stages**	**Delta-Alpha**		**Delta-Low beta**		**Delta-High beta**	
	**Left hand**	**Right band**	**Left hand**	**Right band**	**Left hand**	**Right band**
Entire task	0.0476^*^	0.0131^*^	0.0177^*^	0.0050^**^	0.0501	0.0084^**^
Visual stimulus perception	0.6446	0.1488	0.4006	0.2106	0.4992	0.1421
Mental rotation	0.0129^*^	0.0308^*^	0.0134^*^	0.0268^*^	0.0272^*^	0.0341^*^
Response	0.4311	0.2760	0.4133	0.1644	0.5746	0.1222

* *p < 0.05*;

** *p < 0.01*.

**Table 6:** The *p*-values of clustering coefficient metric of the brain networks based on the OMST topological filtering method in different stages.

**Stages**	**Delta-Alpha**		**Delta-Low beta**		**Delta-High beta**	
	**Left hand**	**Right band**	**Left hand**	**Right band**	**Left hand**	**Right band**
Entire task	0.0030^**^	0.0020^**^	0.0017^**^	0.0016^**^	0.0066^**^	0.0103^*^
Visual stimulus perception	0.0225^*^	0.0053^**^	0.0471^*^	0.0080^**^	0.0305^*^	0.0278^*^
Mental rotation	0.0128^*^	0.0253^*^	0.0102^*^	0.0088^**^	0.0069^**^	0.0475^*^
Response	0.0128^*^	0.0109^*^	0.0255^*^	0.0178^*^	0.0094^**^	0.0078^**^

* *p < 0.05*;

** *p < 0.01*.

**Table 7:** The characteristic path length metric of the brain networks based on two topological filtering methods (setting the threshold as 70% for the manual threshold method).

**Stages**	**Methods**	**Subjects**	**Delta-Alpha**		**Delta-Low beta**		**Delta-High beta**	
			**Left hand**	**Right band**	**Left hand**	**Right band**	**Left hand**	**Right band**
Entire task	Manual	Controls	2.99 ± 0.07	3.00 ± 0.06	3.39 ± 0.07	3.41 ± 0.06	3.69 ± 0.07	3.71 ± 0.08
		Patients	3.10 ± 0.09	3.10 ± 0.07	3.53 ± 0.11	3.51 ± 0.10	3.82 ± 0.13	3.82 ± 0.09
	OMST	Controls	3.75 ± 0.06	3.76 ± 0.06	4.15 ± 0.08	4.17 ± 0.07	4.47 ± 0.09	4.49 ± 0.09
		Patients	3.83 ± 0.10	3.84 ± 0.08	4.28 ± 0.12	4.29 ± 0.10	4.61 ± 0.14	4.62 ± 0.12
Visual stimulus perception	Manual	Controls	2.19 ± 0.03	2.18 ± 0.04	2.47 ± 0.04	2.46 ± 0.06	2.65 ± 0.04	2.64 ± 0.05
		Patients	2.21 ± 0.07	2.23 ± 0.07	2.51 ± 0.08	2.52 ± 0.08	2.69 ± 0.11	2.72 ± 0.11
	OMST	Controls	3.01 ± 0.04	3.00 ± 0.05	3.25 ± 0.06	3.24 ± 0.08	3.43 ± 0.05	3.43 ± 0.06
		Patients	3.00 ± 0.07	3.04 ± 0.08	3.27 ± 0.09	3.28 ± 0.09	3.46 ± 0.11	3.49 ± 0.12
Mental rotation	Manual	Controls	2.50 ± 0.05	2.52 ± 0.05	2.83 ± 0.06	2.85 ± 0.06	3.07 ± 0.07	3.10 ± 0.09
		Patients	2.59 ± 0.07	2.59 ± 0.06	2.93 ± 0.09	2.92 ± 0.06	3.17 ± 0.11	3.16 ± 0.08
	OMST	Controls	3.30 ± 0.05	3.31 ± 0.06	3.61 ± 0.07	3.62 ± 0.07	3.85 ± 0.07	3.87 ± 0.08
		Patients	3.39 ± 0.08	3.38 ± 0.08	3.71 ± 0.09	3.69 ± 0.07	3.96 ± 0.12	3.95 ± 0.07
Response	Manual	Controls	2.15 ± 0.05	2.16 ± 0.04	2.41 ± 0.06	2.42 ± 0.04	2.62 ± 0.09	2.63 ± 0.07
		Patients	2.18 ± 0.07	2.20 ± 0.05	2.46 ± 0.12	2.47 ± 0.06	2.67 ± 0.12	2.71 ± 0.10
	OMST	Controls	2.98 ± 0.06	2.97 ± 0.03	3.22 ± 0.06	3.21 ± 0.03	3.43 ± 0.08	3.42 ± 0.05
		Patients	3.01 ± 0.07	3.00 ± 0.06	3.25 ± 0.12	3.24 ± 0.05	3.46 ± 0.13	3.47 ± 0.07

**Table 8:** The clustering coefficient metric of the brain networks based on two topological filtering methods (setting the threshold as 70% for the manual threshold method).

**Stages**	**Methods**	**Subjects**	**Delta-Alpha**		**Delta-Low beta**		**Delta-High beta**	
			**Left hand**	**Right band**	**Left hand**	**Right band**	**Left hand**	**Right band**
Entire task	Manual	Controls	0.314 ± 0.017	0.313 ± 0.016	0.275 ± 0.013	0.274 ± 0.013	0.258 ± 0.012	0.258 ± 0.013
		Patients	0.293 ± 0.015	0.294 ± 0.015	0.258 ± 0.016	0.259 ± 0.017	0.243 ± 0.016	0.243 ± 0.015
	OMST	Controls	0.328 ± 0.018	0.327 ± 0.018	0.292 ± 0.015	0.293 ± 0.016	0.288 ± 0.016	0.289 ± 0.017
		Patients	0.298 ± 0.015	0.297 ± 0.015	0.264 ± 0.014	0.265 ± 0.014	0.264 ± 0.016	0.265 ± 0.017
Visual stimulus perception	Manual	Controls	0.457 ± 0.012	0.458 ± 0.012	0.385 ± 0.010	0.386 ± 0.012	0.355 ± 0.008	0.357 ± 0.010
		Patients	0.443 ± 0.018	0.441 ± 0.016	0.369 ± 0.010	0.369 ± 0.013	0.342 ± 0.017	0.341 ± 0.011
	OMST	Controls	0.474 ± 0.009	0.474 ± 0.010	0.409 ± 0.012	0.410 ± 0.011	0.400 ± 0.010	0.401 ± 0.009
		Patients	0.461 ± 0.014	0.456 ± 0.015	0.394 ± 0.018	0.390 ± 0.018	0.384 ± 0.018	0.386 ± 0.018
Mental rotation	Manual	Controls	0.383 ± 0.016	0.380 ± 0.014	0.329 ± 0.012	0.328 ± 0.012	0.305 ± 0.011	0.304 ± 0.012
		Patients	0.362 ± 0.016	0.362 ± 0.013	0.313 ± 0.016	0.312 ± 0.015	0.291 ± 0.014	0.290 ± 0.015
	OMST	Controls	0.407 ± 0.016	0.406 ± 0.015	0.355 ± 0.015	0.356 ± 0.015	0.349 ± 0.014	0.349 ± 0.015
		Patients	0.388 ± 0.011	0.390 ± 0.013	0.335 ± 0.014	0.336 ± 0.015	0.331 ± 0.010	0.334 ± 0.016
Response	Manual	Controls	0.482 ± 0.011	0.481 ± 0.006	0.410 ± 0.008	0.408 ± 0.005	0.372 ± 0.008	0.371 ± 0.006
		Patients	0.467 ± 0.011	0.469 ± 0.010	0.393 ± 0.010	0.395 ± 0.010	0.362 ± 0.009	0.361 ± 0.008
	OMST	Controls	0.524 ± 0.007	0.526 ± 0.007	0.466 ± 0.008	0.468 ± 0.006	0.456 ± 0.008	0.456 ± 0.009
		Patients	0.510 ± 0.015	0.516 ± 0.009	0.453 ± 0.015	0.455 ± 0.015	0.445 ± 0.008	0.444 ± 0.008

## 4. Discussion

Focal nerve damage caused by stroke often has a long-distance impact through the residual neural network activities, which causes changes in the brain network (Kawano et al., 2017). Previous research on mental rotation tasks of patients with stroke is mainly limited to intra-frequency brain networks analysis (Yan et al., 2013), this article considers intra-frequency coupling and CFC to explore the impact of stroke. In total, our experiment reveals that CFC presents a new perspective to understand brain activity after stroke.

Several kinds of CFCs are used in research, namely phase to amplitude coupling (PAC), phase to phase coupling (PPC), amplitude to amplitude coupling (AAC) (Cohen, 2008; Dimitriadis et al., 2015; Davoudi et al., 2021a,b). In stroke-related research, some researchers explored PAC. For instance, Yeh et al. (2016) explored muscle activation-movement interaction using PAC based on EMG signals of patients with stroke. But PAC is often limited to single channels and AAC is sensitive to noise. We analyze PPC using the n:m phase synchronization index and construct brain networks by combining intra-frequency and cross-frequency phase coupling. Moreover, a multi-granularity analysis framework is used to extract the important features in temporal and spatial granularity.

In this article, important CFC abnormalities are found through exploring the phase coupling in the whole brain area scale, which can be divided as the coupling between low-frequency and high-frequency or the coupling between low-frequency and low-frequency. As for the coupling between low-frequency and high-frequency, previous research pointed out a mechanism for cognitive control that low frequency (delta and theta) in the prefrontal cortex modulates high-frequency oscillations (beta) (Riddle et al., 2021). Similarly, we have observed the abnormal CFC in delta-low beta and delta-high beta after stroke (Figures 2C,D). As for the coupling between low-frequency and low-frequency, other neurological diseases like Alzheimer's disease existed an abnormal phase coupling phenomenon between different low-frequency bands like delta-alpha (Cai et al., 2018). Our experiment also has observed the unnatural cross-frequency phase coupling of the patients with stroke between low-frequency bands (delta-alpha). Besides, the abnormal cross-frequency phase coupling found in our experiment is highly related to the delta band since low-frequency oscillations like the delta band can reflect injury and recovery of neurons (Cassidy et al., 2020).

This article also explores the information interaction on the scale of the brain hemisphere. Previous studies pointed out that the brain's information communication of the lesion hemisphere and inter-hemisphere in intra-frequency bands like the beta band is significantly affected (Yan et al., 2013), which can also be observed in our experiment (Table 1). Besides, the CFC analysis further reveals that the information interactions of the stroke brain in the lesion hemisphere and inter-hemisphere are affected; more importantly, the information interactions in the non-lesion hemisphere are also affected (Table 2) which can not be observed in intra-frequency bands. CFC is considered to play a crucial role in organizing large-scale networks and cross-distance functional integration (Jirsa and Mueller, 2013). The above findings may illustrate the obstacle of the brain's large-scale information interactions in patients with stroke. In addition, further investigation on critical indicators of CFC is meaningful.

We further found that the information interaction of the delta band in some brain regions is highly affected, like the parietal cortex and central area (Figure 3). To our knowledge, the frontal lobe and central area are in charge of the brain's cognitive and motor function, and the parietal lobe is in charge of spatial information and visual processing. They are also the brain regions highly activated during the motor imagery task for healthy subjects (Kosslyn et al., 1998; Ganis et al., 2000). This finding reveals that functional impairment in these brain regions after stroke may cause poor motor imagery. Besides, previous research found that the stroke brain phase coupling is weaker in beta bands during the mental rotation task (Yan et al., 2013), which is also observed in our experiment (Figure 2). We found the opposite phenomenon: functional connectivity of patients with stroke is significantly higher than that of controls in low beta-high beta (Figure 2D). This phenomenon may be due to the functional regulation of the brain, i.e., this strong cross-frequency coupling is used to compensate for the weak phase coupling in the intra-frequency bands.

Stroke is considered a network disease that changes the whole brain network and its properties (Guggisberg et al., 2019). In our experiment, brain networks constructed by combining intra-frequency and cross-frequency phase coupling are used to explore the functional brain networks in a broader range. Besides local and global graph theoretical metrics are used to analyze the brain networks in temporal granularity, including the entire task stage, visual stimulus perception sub-stage, mental rotation sub-stage, and response sub-stage. The graph theory analysis of the entire task stage reveals that the characteristic path length and clustering coefficient between patients with stroke and controls are significantly different (Figures 4, 5; Tables 5, 6), indicating the stroke brain's weaker functional differentiation and integration ability. The sub-stage analysis found that the graph theoretical metrics between patients with stroke and controls in the mental rotation sub-stage show a stable difference (Tables 3–6). Therefore, the graph theory analysis with CFC facilitates our understanding of the pathological effect of stroke in a broader range.

We apply two topological filtering methods, including the manual threshold and the automated threshold methods. In total, the brain networks created by the two methods consistently reflect the difference between patients with stroke and healthy controls. We further calculate the sparsity of the OMST-based network, and the results are shown in Table 9. In detail, the sparsity of the OMST-based networks in each paired cross-frequency band is in a stable range from 14 to 17%. In addition, the SD of the sparsity of the OMST-based networks are also minor (almost all less than 0.5%). However, the sparsity of brain networks filtered by the manual threshold method is selected as 70%, which is different from the OMST-based brain networks. Compared with the manual threshold method, the OMST topological filtering method removes more connections to create the final “non-redundant” brain networks. Besides, the OMST-based topological filtering method remains both strongly connected edges and weakly connected edges. Therefore, the characteristic path length and clustering coefficient metrics of the OMST-based brain networks are larger than the brain networks based on the manual threshold method shown in Tables 7, 8. Manual thresholding-based brain network analysis is a potential feature selection method for EEG classification tasks (Kong et al., 2018; Huang et al., 2021). In future study, the comparison and evaluation of different topological filtering methods are worth deeply being studied. Besides, the cross-frequency coupling calculation methods such as using mutual information (Tafreshi et al., 2019), transfer entropy (Ahmadi et al., 2020; Xie et al., 2021), and effective feature extraction methods from cross-frequency coupling are also the focus.

**Table 9:** The sparsity (%) of the OMST-based brain networks.

**Stages**	**Subjects**	**Delta-Alpha**		**Delta-Low beta**		**Delta-High beta**	
		**Left hand**	**Right band**	**Left hand**	**Right band**	**Left hand**	**Right band**
Entire task	Controls	15.48 ± 0.16	15.55 ± 0.20	15.08 ± 0.22	15.01 ± 0.27	14.66 ± 0.34	14.69 ± 0.37
	Patients	15.26 ± 0.22	15.37 ± 0.25	14.79 ± 0.39	14.74 ± 0.38	14.24 ± 0.50	14.35 ± 0.52
Visual stimulus perception	Controls	16.91 ± 0.16	16.86 ± 0.19	16.36 ± 0.19	16.36 ± 0.20	16.07 ± 0.17	16.10 ± 0.19
	Patients	16.68 ± 0.21	16.74 ± 0.19	16.05 ± 0.15	16.00 ± 0.17	15.82 ± 0.20	15.90 ± 0.24
Mental rotation	Controls	16.06 ± 0.29	16.07 ± 0.25	15.66 ± 0.25	15.66 ± 0.25	15.34 ± 0.32	15.36 ± 0.32
	Patients	15.88 ± 0.33	15.93 ± 0.39	15.45 ± 0.35	15.58 ± 0.31	15.22 ± 0.42	15.04 ± 0.49
Response	Controls	17.28 ± 0.26	17.35 ± 0.22	16.55 ± 0.23	16.64 ± 0.11	16.24 ± 0.25	16.31 ± 0.17
	Patients	17.03 ± 0.18	17.15 ± 0.22	16.38 ± 0.22	16.56 ± 0.27	16.16 ± 0.29	16.28 ± 0.21

## 5. Conclusion

Evaluating the impact of stroke on brain information interactions is a challenging problem. This article constructs brain networks assembled with intra-frequency and cross-frequency phase coupling based on the EEG data of patients with stroke and healthy controls during the mental rotation task and explores them in temporal and spatial granularity. Through our experiment, the abnormal phase coupling is found in spatial granularity analysis, and the weaker brain functional differentiation and integration ability are observed in temporal granularity analysis in the brain networks of patients with stroke. The brain information interaction of cross-frequency bands is highly affected after a stroke. In total, these findings demonstrate that the coupling between different frequency bands brings a new perspective to understanding the brain's cognitive process after stroke.

## Data Availability Statement

The raw data supporting the conclusions of this article will be made available by the authors, without undue reservation.

## Ethics Statement

Written informed consent was obtained from the individual(s) for the publication of any potentially identifiable images or data included in this article.

## Author Contributions

BR and KY: conception, analysis, and draft. BR, KY, and LZ: analysis and draft. LH: figure. JZ, TQ, and WK: revision. JZ: interpretation. All authors contributed to the article and approved the submitted version.

## Funding

This work was supported by the Key Research and Development Project of Zhejiang Province (2020C04009), National Natural Science Foundation of China (61633010 and U20B2074), Fundamental Research Funds for the Provincial Universities of Zhejiang (GK219909299001-026), National Key Research and Development Project (2017YFE0116800), and Laboratory of Brain Machine Collaborative Intelligence of Zhejiang Province (2020E10010).

## Conflict of Interest

The authors declare that the research was conducted in the absence of any commercial or financial relationships that could be construed as a potential conflict of interest.

## Publisher's Note

All claims expressed in this article are solely those of the authors and do not necessarily represent those of their affiliated organizations, or those of the publisher, the editors and the reviewers. Any product that may be evaluated in this article, or claim that may be made by its manufacturer, is not guaranteed or endorsed by the publisher.
